# Neurotrophic factor-secreting cells restored endogenous hippocampal neurogenesis through the Wnt/β-catenin signaling pathway in AD model mice

**DOI:** 10.1186/s13287-022-03024-6

**Published:** 2022-07-26

**Authors:** Gozal Bahlakeh, Reza Rahbarghazi, Ali Abedelahi, Saeed Sadigh-Eteghad, Mohammad Karimipour

**Affiliations:** 1 Neurosciences Research Center (NSRC), Tabriz University of Medical Sciences, Tabriz, Iran; 2 Stem Cell Research Center, Tabriz University of Medical Sciences, Tabriz, Iran; 3 Department of Applied Cell Sciences, Faculty of Advanced Medical Sciences, Tabriz University of Medical Sciences, Tabriz, Iran; 4 Department of Anatomical Sciences, Faculty of Medicine, Tabriz University of Medical Sciences, Tabriz, Iran

**Keywords:** Neurotrophic factor-secreting cells, Conditioned media, Alzheimer's disease, Neurogenesis, Wnt/β-catenin

## Abstract

**Background:**

Impairment in neurogenesis correlates with memory and  cognitive dysfunction in AD patients. In the recent decade, therapies with stem cell bases are growing and proved to be efficient. This study is a preliminary attempt to explore the impact of NTF-SCs on hippocampal neurogenesis mediated by the Wnt/β-catenin signaling cascade in AD-like mouse brain parenchyma.

**Methods:**

The BALB/c mice were divided into four groups: Control, AD +Vehicle, AD+ TF-SCs-CM and AD+NTF-SCs (*n* = 10). For AD induction, 100 µM Aβ_1-42_ was injected into lateral ventricles. The AD-like model was confirmed via passive avoidance test and Thioflavin-S staining 21 days following Aβ injection. Next, NTF-SCs were differentiated from ADMSCs, and both NTF-SCs and supernatant (NTF-SCs-CM) were injected into the hippocampus after AD confirmation. Endogenous neural stem cells (NSCs) proliferation capacity was assessed after 50 mg/kbW BrdU injection for 4 days using immunofluorescence (IF) staining. The percent of BrdU/Nestin and BrdU/NeuN positive NSCs were calculated. Real-time RT-PCR was used to detect genes related to the Wnt/β-catenin signaling cascade. The spatial learning and memory alternation was evaluated using the Morris water maze (MWM).

**Results:**

Data showed the reduction in escape latency over 5 days in the AD mice compared to the control group. The administration of NTF-SCs and NTF-SCs-CM increased this value compared to the AD-Vehicle group. Both NTF-SCs and NTF-SCs-CM were the potential to reduce the cumulative distance to the platform in AD mice compared to the AD-Vehicle group. The time spent in target quadrants was ameliorated following NTF-SCs and NTF-SCs-CM transplantation followed by an improved MWM performance. IF imaging revealed the increase in BrdU/Nestin^+^ and BrdU/NeuN^+^ in AD mice that received NTF-SCs and NTF-SCs-CM, indicating enhanced neurogenesis. Based on real-time PCR analysis, the expression of PI3K, Akt, MAPK, ERK, Wnt, and β-catenin was upregulated and coincided with the suppression of GSK-3β after injection of NTF-SCs-CM and NTF-SCs. In this study, NTF-SCs had superior effects in AD mice that received NTF-SCs compared to NTF-SCs-CM.

**Conclusions:**

The activation of Wnt/β-catenin pathway via NTF-SCs can be touted as a possible therapeutic approach to restore neurogenesis in AD mice.

## Background

Neurodegenerative disorders such as Alzheimer's disease (AD), Parkinson's disease, and Huntington's disease exert an extensive financial burden on the health system [1]. AD is known as the common age-related dementia without efficient treatments [2–4]. From pathological aspects, AD is diagnosed by the formation of Aβ senile plaques, and neurofibrillary tangles (NFTs) consisting of hyperphosphorylated Tau proteins which contributed to cognitive impairment and functional deficits. [5–7]. The hippocampus is one of the two main neurogenic niches in the adult brain. The phenomenon of hippocampal neurogenesis is done by the promotion of neural stem cells (NSCs) proliferation in the subgranular zone (SGZ), which subsequently differentiate into mature neurons in the dentate gyrus (DG) [8].

It is thought that the SGZ neurogenesis is interrupted following the onset of AD, affecting the memory function and cognition capacity [9, 10]. Further, the formation of Aβ deposits modulates various cascades inside the brain parenchyma, leading to suppression of neurotrophic factor signaling and neurogenesis decline [11]. Neurotrophic factors are a group of secreted proteins that exert numerous roles in neural and even non-neural tissues. It was reported that these proteins reduce the degenerative process in neurodegenerative disorders [12]. The neurotrophin family contributes to several neurogenesis-related processes, for example, neuronal proliferation, differentiation, maturation, neurite expansion, and synaptic plasticity [13]. Among different growth factors, nerve growth factor (NGF), expressed in the hippocampus and brain cortex, is crucial for the cholinergic system function. It is thought that this factor can induce survival, growth, neuronal differentiation, and migration of NSCs [12, 14]. The NGF regulates amyloid peptides production through the amyloidogenic pathway [12]. Along with NGF, the brain-derived neurotrophic factor (BDNF) is the most abundant neurotrophin within the brain parenchyma. The attachment of this factor to its receptor can improve cognitive and memory functions by promoting neurogenesis in the hippocampus [15]. Certain downstream effectors such as MAPK/ERK1/2 and PI3K/AKT signaling pathways can be modulated in response to these factors [16]. MAPK/ERK pathway links extracellular and intracellular signaling pathways and regulates cell bioactivities such as proliferation, differentiation, migration, and apoptosis [17]. The activation of AKT leads to the regulation of multiple pathways associated with proliferation, survival, metabolism, vesicle, and glucose transportations [18]. Studies have reported that Aβ deposition dysregulates the normal activity of ERK/MAPK and PI3K/AKT axes and consequently hindered the migration of NSCs and causes neuron cell death [4]. Moreover, several findings have shown that the glycogen synthase kinase-3β (GSK-3β) can be effected via PI3K/AKT signaling [19]. Among various kinases, GSK-3β is the main kinase that dysregulates Tau phosphorylation and is accumulated following AD [5, 20]. The activation of GSK-3β increases β-catenin phosphorylation, leading to its inhibition [19]. Besides, it was suggested that Wnt/β-catenin signaling regulates several pathways like adult hippocampal neurogenesis, cell migration, survival, synaptic plasticity, and cognition [21, 22]. The production and accumulation of Aβ_1-42_ are reduced which coincided with NFT (neurofibrillary tau tangles) formation [20, 23]. AD-like condition reduces the Wnt secretion of astrocytes and inhibits the Wnt/ β-catenin signaling. Further, AD pathogenesis disturbs this signaling pathway hence the neurogenesis is impeded [24].

The discovery of different stem cell types has revolutionized human medicine and is touted as an alternative approach to cure several pathologies. Among several types of stem cells, adipose tissue-derived stem cells can be differentiated into neurotrophic factor-secreting cells (NTF-SCs) with the ability to produce and secret neurotrophic factors like BDNF and NGF [25, 26]. These cells have the advantage of transplantation and, as a vehicle that can deliver NTFs to the transplanted area further, have migratory ability to the injured regions [27]. The NTF-SCs have shown to be a valuable treatment in the AD in vitro model [26]. Since altered neurogenesis is a manifestation of AD, the therapy with the potency of neurogenesis enhancement seems favorable. Further, The SGZ neurogenesis has shown to be modulated with local cells' signals like astrocytes and endothelial cells [28]. Thus in this study, we investigated the NTF-SCs' therapeutic effects on the improvement of endogenous neurogenesis, the associated special memory, and the Wnt/β catenin signaling pathway in an AD model of mice.

## Materials and methods

### Animals and ethics statement

All experiments and analyses were approved by the local ethic committee of the Tabriz University of Medical Sciences (NO. IR.TBZMED.VCR.REC.1397.444). In this study, 40 male BALB/c, weighing 25–30 g, were obtained from the laboratory animal care center of Tabriz University of Medical Sciences. Five mice were maintained in standard plastic cages. All the conditions of the room, including temperature (23 ± 1 °C), light (12:12 light/dark cycle), and humidity (50 ± 5%), were controlled at the laboratory of Neurosciences Research Center of Tabriz University of Medical sciences.

### Isolation, expansion, and characterization of ADMSCs

Adipose tissues were collected from the inguinal region of mice and washed completely with PBS to clear the blood. In brief, the tissues were dissected with a sterile surgical knife, and then digested for 30 min in PBS solution containing 2% bovine serum albumin (BSA) and type I collagenase (2 mg/ml). The collagenase was inactivated via DMEM/F12 supplemented with 10% fetal bovine serum (FBS). The procedure was centrifuged with the centrifugation of cell suspension (1200 rpm for 5 min. The isolated cells were collected and cultured in DMEM/F12 with FBS and 1% Penicillin/Streptomycin. After 24 h, the floating cells were removed, and the adherent cells were sub-cultured at 70–80% confluence. For flow cytometric characterization**,** cells at passage 3 were trypsinized and washed twice with 1% PBS/BSA. Then, cells were incubated with CD105 (ab156756, Abcam), CD90 (sc-53116, Santa Cruz Biotechnology), and CD45 (ab10558, Abcam) for one hour at room temperature and washed in PBS. For labeling, FITC-conjugated secondary antibody was added and samples were kept in the dark for one hour. Samples were fixed in a 1% paraformaldehyde (PFA) solution. Using FACSCalibur® system and FlowJo software (version 7.6.1) cells were analyzed.

### NTF-SCs differentiation of ADMSCs

The differentiation of ADMSCs to NTF-SCs was inducted according to previous protocols [29]. ADMSCs at passage 3 were cultured in appropriate media to induce differentiation toward NTF-SCs in two distinct steps. First, cells were incubated in DMEM/F12 culture medium with 20 ng/mL basic fibroblast growth factor (bFGF), 20 ng/mL epidermal growth factor (EGF), and N2 supplement. After 72 h, the culture medium was replaced with a medium supplemented with 0.5 mM Isobutylmethylxanthyle (IBMX), 1 mM dibutyryl cyclic AMP, 5 ng/ml platelet-derived growth factor (PDGF), 20 ng/ml bFGF, and 5 ng/ml human neuregulin 1-B1/HRG1-B1 EGF domain. To affirm the NTF-SC-like phenotype, the cells were immune-stained with anti-glial fibrillary acidic protein (GFAP), and the secretion ability was assessed via western blot for released BDNF and NGF proteins.

### Immunofluorescence staining

For immunofluorescence staining, the cells were fixed with 4% PFA solution for 20 min and permeabilized using 0.1% Triton-X-100 for 5 min. Then, cells were blocked using 3% BSA and incubated with anti-GFAP (ab7260, Abcam) antibody for one hour. After PBS wash, cells were incubated with FITC-labeled secondary antibody (ab6787, Abcam) for 60 min in RT. For nuclear counterstaining, the slides were exposed to 1 µg/ml 4′,6-diamidino-2-phenylindole (DAPI), and stained cells were visualized using an invert fluorescence microscopy. The percentage of GFAP^+^ was calculated according to the following formula GFAP^+^  + DAPI^+^ cells/DAPI^+^ cells × 100. The assessment was performed three times.

### Western blotting

In short, the supernatant of the NTF-SCs was collected and protein concentration measured using the Lowry protein method. Using 12% SDS-PAGE electrophoresis, proteins were separated and electro-transferred to the polyvinylidene fluoride (PVDF) membrane. The membrane was blocked with TBST supplemented with 5% skim milk followed by incubation with primary antibodies against BDNF (ab203573, Abcam), NGF (ab6199, Abcam), and GAPDH (ab181602, Abcam) overnight at 4 °C. The membranes were rinsed in TBST three times (each for 5 min). Then, HRP-conjugated secondary antibodies were used (60 min at RT). After three-time PBS washes, immune-reactive bands were detected using the ECL kit (RPN3243).

### Experimental procedures

The animals were divided into Control, AD + vehicle, AD + NTF-SCs-CM and AD + NTF-SCs groups (*n* = 10). In this study, control mice received no manipulation. In the AD + vehicle group, 5 µl basic DMEM/F12 was used as a vehicle. Mice in the AD + NTF-SCs-CM group received 5 µl conditioned medium (CM), while in the NTF-SCs group, about 25 × 10^4^ NTF-SCs were re-suspended in 5-µl basic DMEM/F12 and transplanted. All groups except the control group received intracerebroventricularly (ICV) 100 µM Aβ_1-42_ for modeling the AD via stereotaxic surgery. To prepare a stock solution, 500 μg Aβ_1-42_ (ab120959, Abcam) was diluted in 500 μl ammonium hydroxide 1% (NH_4_OH). Five days before injection, the Aβ1-42 solution was aggregated at 37 °C inside a water bath. The mice were anesthetized via 4% isoflurane, and their skull was fixed in stereotaxic apparatus. The mice were kept anesthetized during the surgery with 30% O_2_, 70% nitrous oxide, and 1.5% isoflurane. The body temperature was monitored with the rectal sensor of the heating pad, and the eyes were protected from dryness via antibacterial ophthalmic ointment. After shaving and swapping, a 1-cm long incision was applied on the scalp. A hole was opened by drilling the skull, and a 26-gauge sterile guide cannula was inserted into the injection site. On basis of the Franklin and Paxinos mouse brain atlas, the following coordinates from the bregma were utilized for ICV injection of amyloid-beta 1–42 and/or hippocampal infusion of NTF-SCs, NTF-SCs-CM, and DMEM via infusion pump, respectively: anteroposterior (AP) = -0.2, mediolateral (ML) =  + 1 , and dorsoventral (DV) = − 2.5 and AP = − 1.8, ML =  + 0.75 and DV = − 2.2. The reflux was avoided by an additional 5 min keeping of the cannula in the injection site. Then, the injection cannula was gently retracted. The incision was sutured, and the animal was monitored in a warm place until it spontaneously moved. Twenty-one days following the Aβ injection, the mice were assessed via the step-through latency to monitor learning potential and memory capacity. Then, 5-µl DMEM/F12, NTF-SCs-CM, or NTF-SCs at a velocity rate of 1 µl/minute were injected into the hippocampus using an infusion pump [30].

### Passive avoidance task (shuttle box test)

For evaluation of the learning and memory in the feedback to a stress stimulator, the shuttle box apparatus was utilized. The apparatus has two boxes, the light, and the dark ones. The boxes are connected through a guillotine door. The dark box has a floor of stainless steel bars. The test has three steps: habituation, training, and probe. In the habituation phase, the mice were adapted to the box. In the training phase, the mouse was gently placed in a light box and one minute later, the door was opened. When the mouse moves in a dark box, immediately obtained the 40 V, 0.5A, 2 S electric shock. On probe day, each mouse was gradually placed in the light chamber, and the latency time as the step-through latency was recorded. The memory retention cut-off time-limited to 5 min [31].

### Thioflavin-S staining

The Thioflavin-S staining was used for the detection of Aβ plaques. Before the staining, the paraffin-embedded sections were deparaffinized and hydrated with xylene and a series of reducing ethanol (100, 95, 70, and 50). The ethanol was rinsed with tap water. Then, 1% Thioflavin-S was applied to stain the sections in a dark place for 10 min [32]. The slides were washed in 70% ethanol and distilled water and cover-slipped. Finally, a fluorescence microscope was utilized to identify plaques.

### NTF-SCs labeling

NTF-SCs were labeled utilizing Cell Tracker™ CM-DiI fluorescent Dye. In this regard, the medium removed NTF-SCs were incubated in 20-μM dye solution for 20 min inside the incubator. Then, the cells were washed using PBS three times to eliminate unbound dye and prepare the cells for injection.

### BrdU administration

For the characterization of cells in the mitotic phase, the BrdU (thymidine analog) labeling is a routine cell tracer that can integrate into S-phase DNA. The BrdU can be recruited along with endogenous neuronal markers, Nestin and NeuN. In this regard, BrdU (ab142567, Abcam) was diluted in normal saline at 0.09% for obtaining a 10 mg/ml concentration. Then, the lower abdominal region was disinfected, and BrdU solution (50 mg/kg) was infused via intraperitoneal (IP) injection. An injection was given per day and started one day after treatment (NTF-SCs transplantation or NTF-SCs-CM/DMEM injections) for four continuous days to label more dividing cells. The brains for BrdU/Nestin investigation and the brains for BrdU/NeuN were harvested one or 35 days after the last BrdU injection, respectively.

### Morris water maze (MWM) test

Special learning and memory functions were examined by the Morris water maze [33]. Briefly, the circulating pool was filled with water. The room's static clues were covered via black curtains. The pool was divided into four quadrants including southeast (SE), southwest (SW), northwest (NW), and northeast (NE). The transplant platform, which should be about 1 cm above (for the visible platform stage) or under the water (for the hidden platform stage), was placed in one of the quadrants. The NW was chosen as the test initiation position. There was a camera above the MWM for recording the animal swimming. In the visible platform stage, a beacon labeled the platform then the released mouse had to find the platform inside the pool for 60 s, and the time until the mouse reached the platform was recorded. If the mouse could not reach the platform within 60 s, it was directed to the platform and the escape latency time was reported as 60 s. Each mouse was given 4 trials per day. In the hidden stage, the platform was fixed and hidden under 1 cm water. For five continuous days, each mouse was trialed 4 times per day at 10 min intervals and the escape latency time was recorded (the spent time to find the platform). On the probe day, one day after the learning trial, the platform was taken away and the mouse was released into the pool for 60 s. The spent time in the target quadrant was noted and analyzed.

### Double immunofluorescence staining of brain tissue

Using IP injection of mixed ketamine/Xylazine (10/100 mg/kg), mice were euthanized, and brain samples were fixed in 4% PFA buffer. To monitor endogenous neurogenesis, we performed BrdU/Nestin (endogenous NSC proliferation) and BrdU/NeuN (endogenous NSC maturation) staining one and 35 days after the last BrdU injection, respectively. To show whether transplanted NTF-SCs can proliferate and differentiate into the neural lineage, we also monitored the levels of the above-mentioned factors in Dil-labeled cells one and 35 days after NTF-SCs-CM and/or NTF-SCs injection. In brief, coronal sections were placed on poly-L-lysine coated slides, deparaffinized and hydrated in Xylazine and descending ethanol solutions, respectively. After 10 min of incubation in TBS buffer, antigen retrieval was done using 10-mM sodium citrate buffer for 10 min at 100 °C. The procedure was continued with the incubation of slides in 2 N HCL (37 °C for 30 min) to denature DNA and increase available epitopes for the anti-BrdU antibody. The acid was neutralized through incubation in 0.1 M boric acid for 10 min followed by 3 times rinsing via TBS at RT. After that the sections were incubated in a TBS solution containing 3% goat serum plus 0.3% Triton-X 100 for 30 min to decrease the non-specific bindings. Then, the slides were exposed to a mixture of primary antibodies (mouse monoclonal anti-Nestin antibody, Clone: 10C2, Cat#: MA1-110, Dilution: 1:100, and mouse monoclonal anti-BrdU antibody, Clone: ZBU30, Cat#: 03–390, Dilution: 1:100) to evaluation of the proliferation and (mouse anti-NeuN Millipore, clone A60, cat#: MAB377, Dilution: 1:100 and mouse monoclonal anti-BrdU antibody, Clone: ZBU30, Cat#: 03–390, Dilution: 1:100) to evaluation of maturation over nightly in 4 °C. Then, the slides were rinsed and incubated in a mixture of secondary antibodies (Alexa Fluor 594 conjugated goat anti-mouse secondary antibody, Cat # A-11032, Dilution: 1:1000 and Alexa Fluor 488 conjugated goat anti-mouse secondary antibody, Cat # A28175, Dilution: 1:2000) in a dark wet place at RT for 1 h. The TBS-washed slides were counterstained using DAPI (Sigma-Aldrich). Then co-labeled slides were washed in TBS, and cover-slipped. The sections were observed by LaboMed fluorescence microscope. The BrdU/Nestin and BrdU/NeuN positive cells were counted in the 100 BrdU-positive cells and presented as a percentage. To track and monitor NTF-SC activity, 5-μm-thick sections were incubated with anti-Nestin and -NeuN antibodies and FTIC-labeled antibodies as above-mentioned. Nuclei were stained using DAPI and the percent of Dil/Nestin and Dil/NeuN positive cells were visualized and counted under fluorescence microscopy.

### Real-time PCR analysis

In brief, Akt, Erk1/2, PI3K, MAPK, Wnt3a, β-Catenin, and GSK-3β expression were assessed in different groups. To this end, total RNA contents were isolated from brain samples using the RNeasy mini-kit (Qiagen). After that, cDNA was synthesized using the Revert AidTM First Strand cDNA Synthesis kit (Fermentas). The real-time PCR reaction was done using Maxima™SYBR Green/ROX qPCR Master Mix (2X) kit (Fermentas). The expression of each gene was quantified using the 2^−ΔΔCT^ formula. This assay was done in triplicate. The primers were designed using Oligo7 software (Table 1).

**Table 1:** Primer sequence

Gene	Primer	TM (˚C)
β-actin	F: GGCTGTATTCCCCTCCATCG R: CCAGTTGGTAACAATGCCATGT	60
Gsk3b	F: GAGCCACTGATTACACGTCCAG R: CCAACTGATCCACACCACTGTC	63
Wnt3a	F: CACCACCGTCAGCAACAGCC R: AGGAGCGTGTCACTGCGAAAG	58
Akt1	F: GCTTCTATGGTGCGGAGATTGT R: CGTCCTTGATCCCCTCCTTG	63
MAPK1 or ERK2	F: ATTGATATTTGGTCTGTGGGCT R: CCTGTTCCATGGCACCTTATTT	63
MAPK3 or ERK1	F: TCTGGTCTGTGGGCTGCATTC R: GGTAGTTTCGGGCCTTCATGT	63
PI3K	F: AGTAACAGACTAGCCAGAGACAA R: TTGACAGACAGAAGCAATTTGGG	60

### Statistical analysis

The Graph Pad Prism software was utilized for statistical analysis. The data were analyzed by one-way ANOVA. The statistical significance was considered *p* < 0.05.

## Results

### ADMSCs characterization

**Fig. 1:**
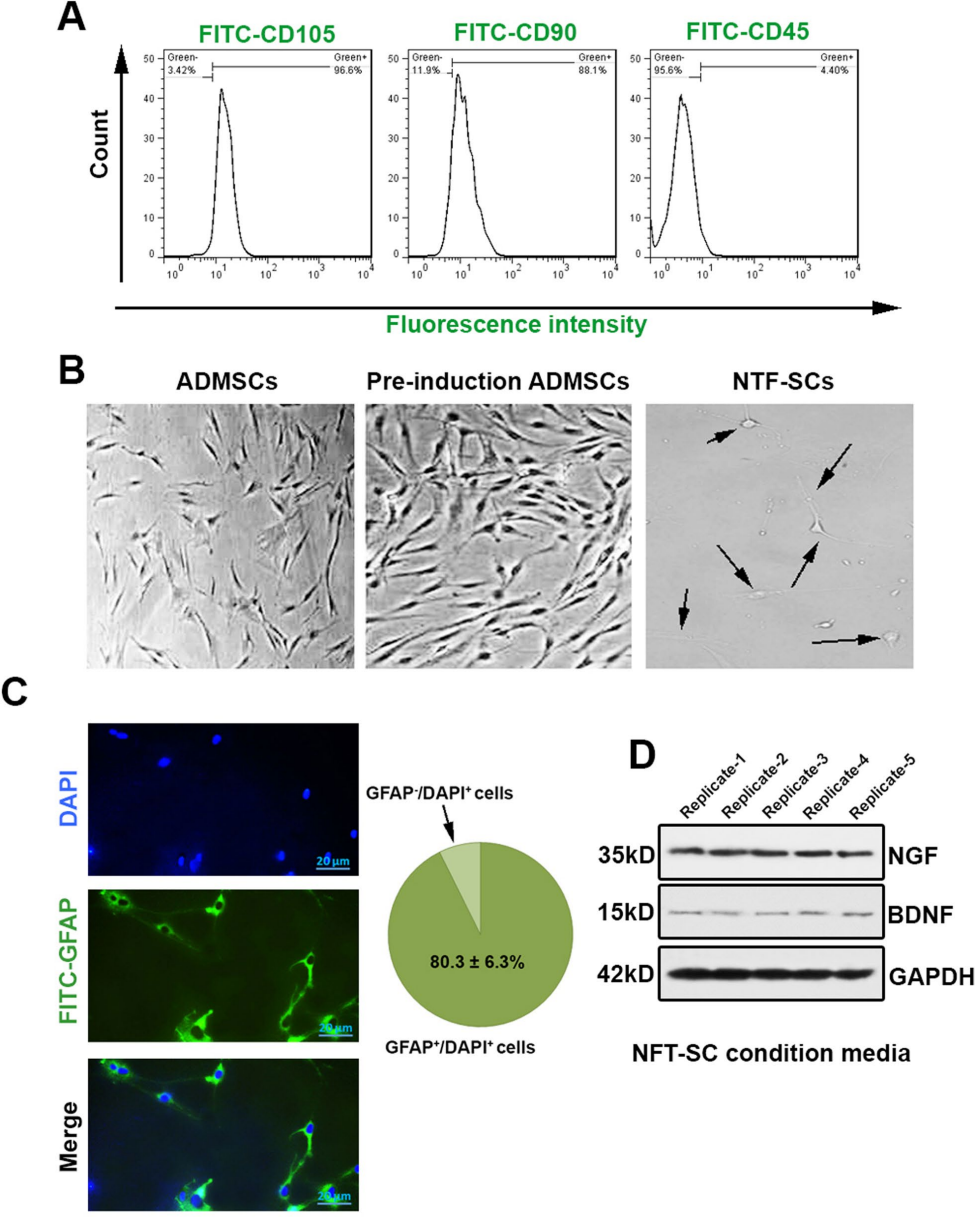
NTF-SCs differentiation and characterization (**A–D**). **A** Flow cytometry histograms of three passaged ADMSCs for (CD105, CD90) were positive and negative for (CD45), indicating the ability to express of ADMSCs surface markers by isolated cells. **B** Bright-field imaging of ADMSCs, pre-induction ADMSCs (third passage) and NTF-SCs cultures showed the expanded ADMSCs have a fibroblast-like phenotype and the NTF-SCs with an astrocyte-like appearance. **C** Immunohistochemistry imaging for GFAP astrocyte cells marker and DAPI staining for the nucleus of the cells demonstrated that 80.3% of the differentiated cells expressed the GFAP marker. **D** In Western blot analysis for NTF secretion in NTF-SCs-CM, the blots were developed with NGF and BDNF antibodies, and an average of 35 KDa and 15 KDa of NGF and BDNF were detected, respectively. One-way ANOVA and Tukey post hoc analysis. *p < 0.05; **p < 0.01; ***p < 0.001; and ****p < 0.0001. NTF-SCs, Neurotrophic-factor-secreting astrocyte-like cells. ADMSCs, Adipose-derived mesenchymal stem cells. NTF, Neurotrophic factor, CM, Culture medium

Flow cytometry analysis showed that the isolated cells were positive for stem cell markers such as CD105 (96.6%), and CD90 (88.1%), whereas about the percent of CD45^+^ cells reached 4.5%. These features showed the suitability of our protocol in the isolation of ADMSCs (Fig. 1A). Bright-field microscopy revealed that the isolated cells had fibroblast-like and spindle shape morphology (Fig. 1B).

### NTF-SCs differentiation of ADMSCs

GFAP staining confirmed the existence of astrocyte-like morphology (Fig. 1C). Based on the semi-quantitative analysis, we noted that about 80.3 ± 6.3% of cells are double GFAP/DAPI positive, indicating an appropriate orientation of ADMSCs toward NTF-SCs.

### Differentiated NTF-SCs can release NGF and BDNF

To examine the paracrine activity of differentiated NTF-SCs, we monitored the contents of NGF and BDNF in the supernatant using western blotting (Fig. 1D). Data showed that the NTF-SCs could secrete notable amounts of NGF and BDNF to the supernatants (Fig. 1D). The data indicated that differentiated NTF-SCs are eligible to produce certain neurotrophic factors which are important to neuronal activity in a paracrine manner.

### Aβ_1-42_ injection impaired learning capacity and memory function

**Fig. 2:**
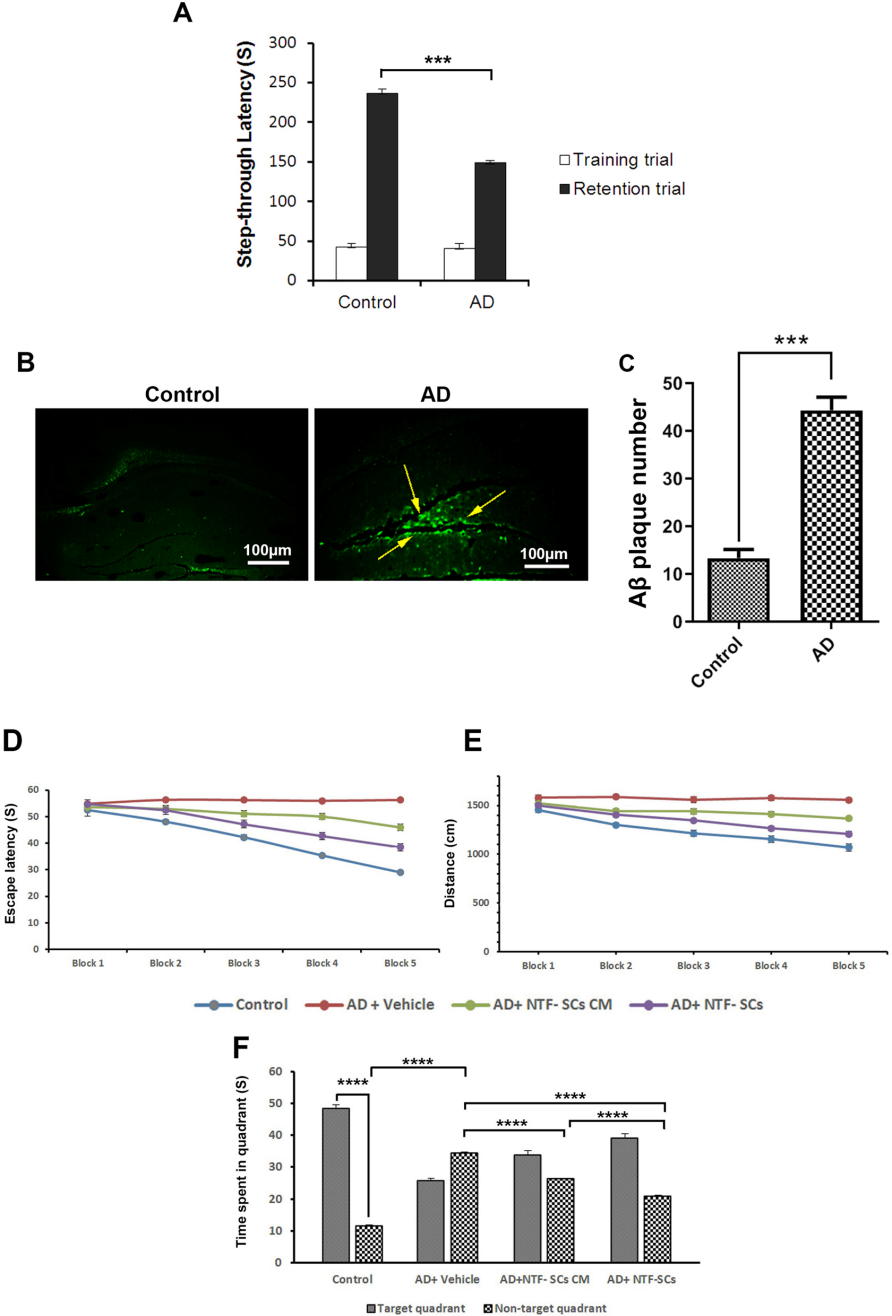
Aβ_1-42_-induced memory impairment and senile plaques (**A–C**). Passive avoidance test (**A**). Evaluating the learning and memory following Aβ 1–42 injection demonstrated that the step-through latency assessment during the retention trial decreased in the AD mice compared to the control group (*p* < 0.001). Thioflavin-S staining (**B–C**). Histologic data showed that the number of senile plaques was enhanced in the AD + vehicle group in comparison with the control one (*p* < 0.001). One-way ANOVA and Tukey post hoc analysis. *p < 0.05; **p < 0.01; ***p < 0.001; and ****p < 0.0001. AD, Alzheimer’s disease. Morris water maze test (**D–F**). NTF-SCs transplantation decreased the escape latency over 5 days compared to the AD + Vehicle group (**D**). NTF-SCs were the potential to reduce the cumulative distance to the platform in AD mice compared to the AD + Vehicle group (**E**). Moreover, the time spent in target quadrants was reduced in AD mice and transplantation of NTF-SCs ameliorated the Aβ 1–42 adverse effects (**F**)

The passive avoidance test was used to assess learning capacity and memory function (Fig. 2A; *p* < 0.05). Based on data, analyses revealed different duration times of step-through latency in the probe phase (21 days after the AD induction) compared to the control group (*p* < 0.001). Aβ_1-42_ administration significantly reduced the step-through latency time in the AD group. These results represented that aggregated Aβ_1-42_ could reduce the learning ability and memory retrieval.

### Aβ_1-42_ injection increased amyloid plaques in the brain parenchyma

To detect the formation of amyloid plaques inside brain parenchyma, we performed Thioflavin-S staining (Fig. 2B–C). Data indicated amyloid plaques in the hippocampus of mice received 100 µM Aβ_1-42_. According to our data, we found an average amyloid plaque number of 13.33 and 44.37 in the control and AD groups, respectively (*p* < 0.001; Fig. 2B–C). Taken together, it was shown that direct injection of Aβ_1-42_ into the intra-cerebroventricular region can promote amyloid plaque formation in the hippocampus, mimicking AD-like features.

### NTF-SCs transplantation improved the learning and memory performance

To assess the spatial learning and memory following the NTF-SCs transplantation, the MWM test was used (Fig. 2D–F). According to our data, significant statistical differences were notified among experimental groups in escape through latency and swim distance to find the platform in the training phase (*p* < 0.05). In the control, NTF-SCs-CM, and NTF-SCs groups a significant reduction was found in escape latency and swim distance compared to the AD + vehicle group during the training phase. The statistical data of probe day showed that the mean time spent in the target quadrant significantly enhanced in the control, NTF-SCs-CM, and NTF-SCs groups relative to the AD + vehicle group (*p* < 0.0001; Fig. 2D–F). The result of this study suggests that the NTF-SCs could improve learning and memory.

### NTF-SCs transplantation increased the adult endogenous hippocampal neurogenesis

**Fig. 3:**
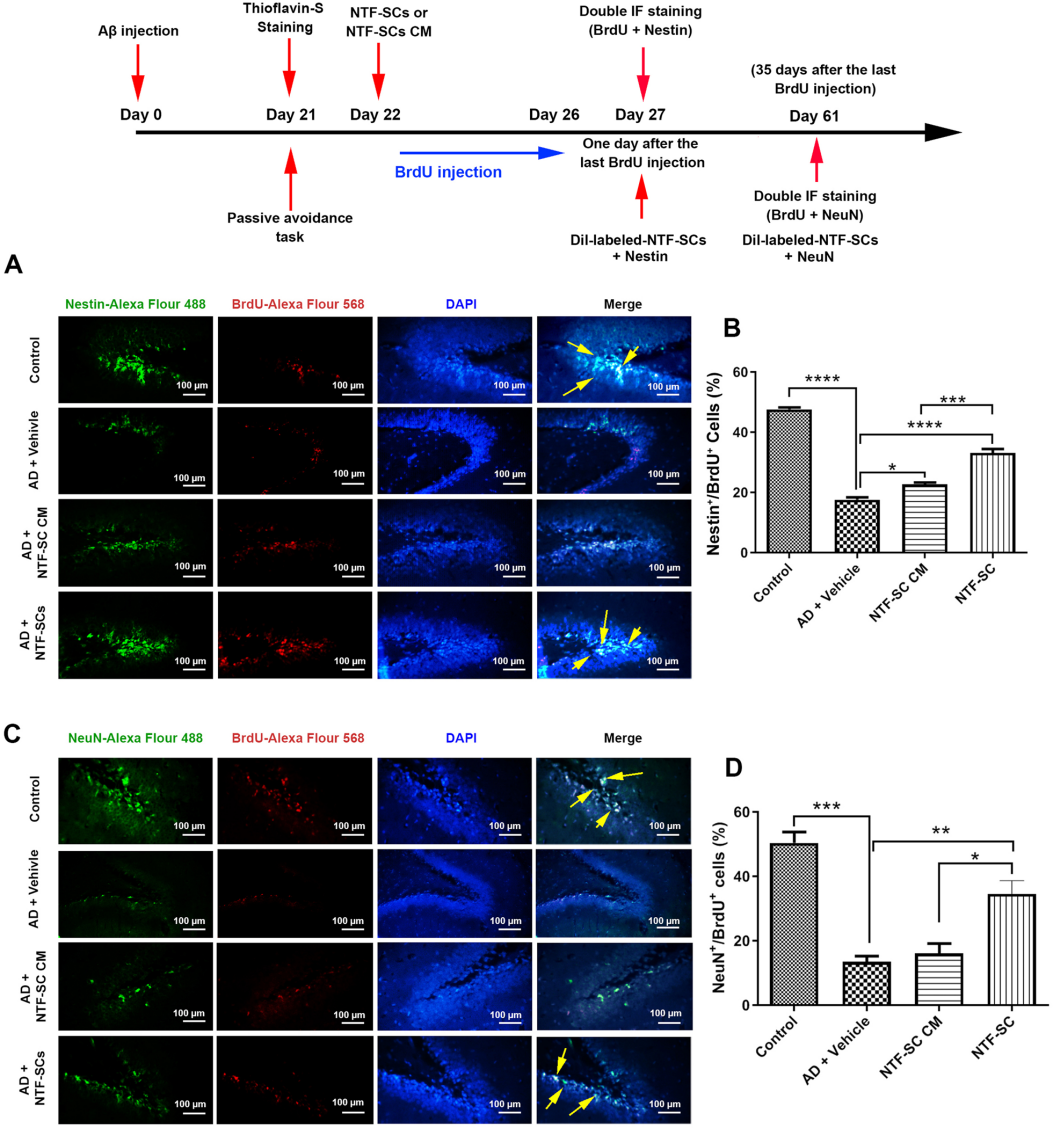
Schematic illustration of the current experimental design and Immunofluorescence imaging for endogenous neurogenesis (**A–D**). IF imaging and semi-quantitative analysis of BrdU/Nestin positive cells one day after the last BrdU injection (**A–B**). The percent of double-labeled BrdU/Nestin positive cells was reduced significantly in the AD + Vehicle group compared to the control and other groups. Compared to the NTF-SC CM group, direct injection of NTF-SC transplantation had the superiority to increase the percent of double-labeled BrdU/Nestin positive cells in the hippocampus of mice subjected to AD-like conditions. IF imaging and semi-quantitative analysis of BrdU/NeuN positive cells 35 days after the last BrdU injection (**C–D**). According to our data, the percent of double-labeled BrdU/Nestin positive cells was reduced significantly in the AD + Vehicle group compared to the control and other groups. The injection of NTF-SC increased the percent of double-labeled BrdU/ NeuN positive cells more than that of the NTF-SC CM group. These data represent the enhanced proliferation and neurogenesis of endogenous NSCs in groups that received NTF-SCs one and 35 days after the last BrdU injection in comparison with the AD + vehicle group. One-way ANOVA and Tukey post hoc analysis. One-way ANOVA and Tukey post hoc analysis. *p < 0.05; **p < 0.01; ***p < 0.001; and ****p < 0.0001

To explore the effects of NTF-SCs transplantation on neurogenesis, survival, and maturation of endogenous NSCs were evaluated in the brain tissue samples via IF staining (Fig. 3A–D). In our experiment, we did the BrdU/Nestin and BrdU/NeuN double staining on 1 or 35 days after the last BrdU infusion, respectively. Our data revealed that the number of BrdU/Nestin and BrdU/NeuN positive cells was significantly reduced in AD + vehicle mice related to the control group (*p* < 0.05; Fig. 3A–D). In contrast, the NTF-SCs transplantation could significantly enhance the BrdU/Nestin^+^ cells and BrdU/NeuN^+^ cells in comparison with the AD + vehicle group (Fig. 3A–F). Taken together, our results suggested that transplanted NTF-SCs were stimulators for neurogenesis in SGZ of the hippocampus despite the presence of AD-like pathology.

### NTF-SCs differentiated into mature neurons following transplantation

**Fig. 4:**
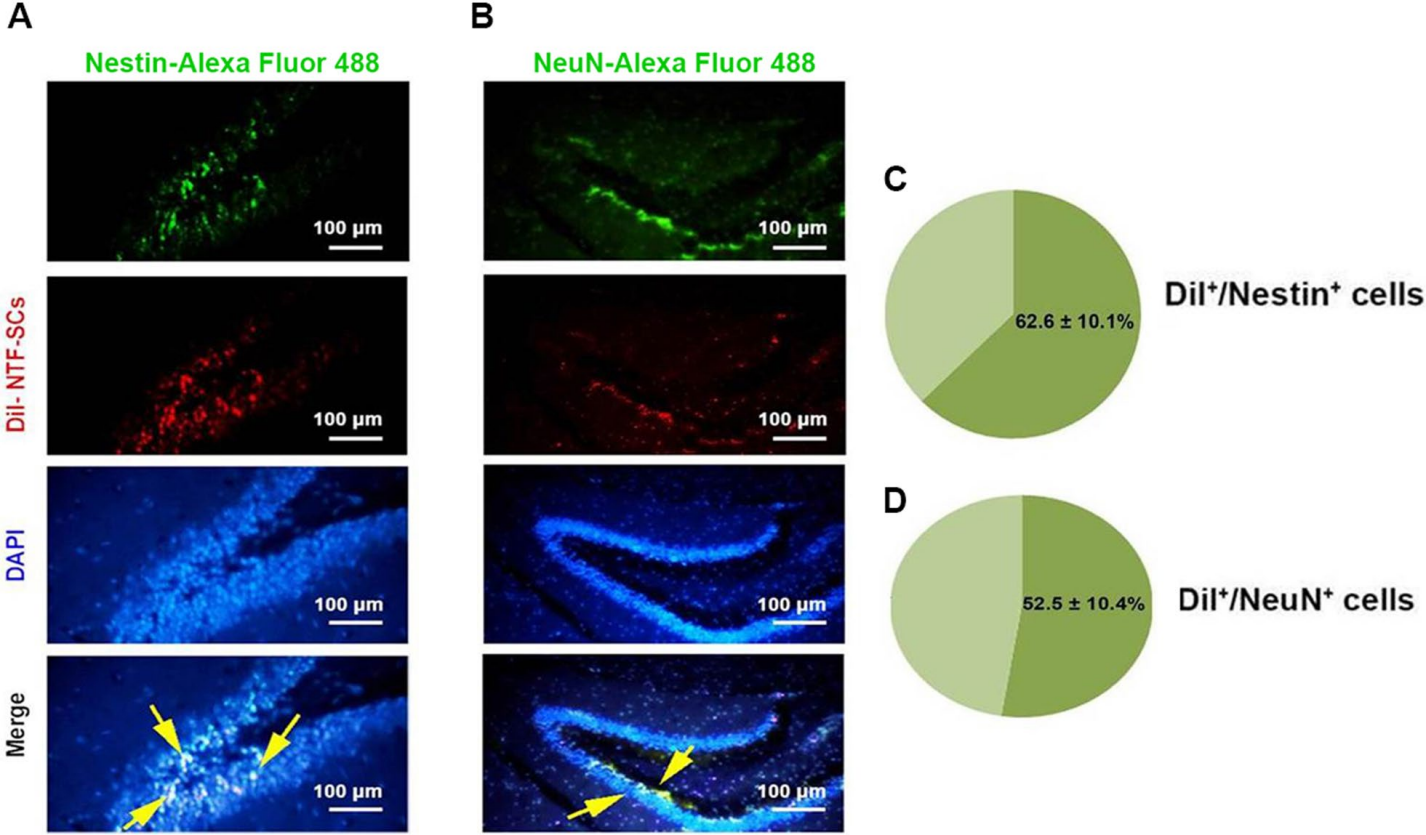
Immunofluorescence imaging for Dil-labeled NTF-SCs (**A–D**). Monitoring the potency of Dil-labeled NTF-SCs to express nestin marker one day after transplantation into the DG (**A**). Differentiation of Dil-labeled NTF-SCs into mature NeuN.^+^ neurons 35 days after cell transplantation (**B**). Assessment of the NTF-SCs in the SGZ niche suggested that 62.6% and 52.5% of the labeled cells were positive for double Dil/Nestin (**C**) and Dil/NeuN (**D**), respectively. One-way ANOVA and Tukey post hoc analysis. *p < 0.05; **p < 0.01; and ***p < 0.001

To evaluate the effect of the SGZ niche on NTF-SCs proliferation and differentiation, we performed an IF analysis (Fig. 4). Results represented that about 62.2% of Dil-labeled NTF-SCs expressed Nestin 5 days after transplantation (Fig. 4). The double IF staining showed about 52.5% of the Dil-labeled NTF-SCs expressed NeuN 35 days following transplantation (Fig. 4D). These data suggest that transplanted NTF-SCs, known as astrocyte-like cells, could proliferate and become mature neurons within the SGZ niche.

### NTF-SCs transplantation regulated the expression of Wnt/β-catenin signaling pathway genes

**Fig. 5:**
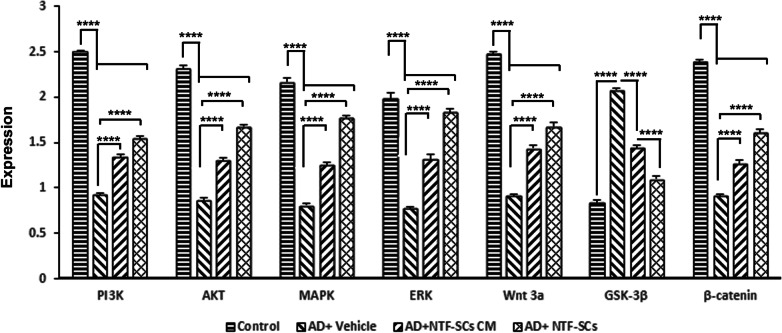
Real-time RT-PCR analysis for the Wnt/β catenin signaling pathway. NTF-SCs transplantation increased the expression levels of the *PI3K*, *Akt*, *MAPK*, *Erk*, *Wnt3a*, and *β-Catenin*, while the *GSK3β* level was decreased. One-way ANOVA and Tukey post hoc analysis. *p < 0.05; **p < 0.01; ***p < 0.001; and ****p < 0.0001

Real-time PCR displayed that NTF-SCs transplantation could regulate the expression of the PI3K, Akt, MAPK, ERK, Wnt3a, GSK-3β, and β-catenin genes in the mouse hippocampus following AD (Fig. 5). The promotion of AD-like conditions can reduce the expression of PI3K, Akt, MAPK, ERK, Wnt3a and β-catenin compared to the control group (*p* < 0.05; Fig. 5). Our results showed that the expression of Akt, Erk1/2, PI3K, MAPK, Wnt3a, and β-catenin genes wad upregulated in AD + NTF-SCs, AD + NTF-SCs-CM groups compared to the AD + vehicle group (Fig. 5). These effects were more evident in groups that received NTF-SCs-CM (*p* < 0.05). However, the GSK-3β expression level was escalated in the AD + vehicle group compared to the control group (*p* < 0.05). The injection of NTF-SCs and NTF-SCs-CM can return GSK-3β expression to near-to-normal levels. These effects were more evident in the AD + NTF-SCs-CM group. Taken together, this study indicated that NTF-SCs-CM and NTF-SCs could reduce the GSK-3β level in AD mice which is in contrast to the up-regulation of PI3K, Akt, MAPK, ERK, Wnt3a, and β-catenin.

## Discussion

AD as the most common form of dementia is a multifactorial disease. The growing aging population due to the long life escalates the risk of chronic diseases and subsequent physical, mental and cognitive disabilities. Therefore, finding a strategy for treatment seems urgent and challenging. The atrophy of the hippocampus and impaired neurogenesis are demonstrated as important hallmarks of AD [34, 35]. Following AD initiation, the accumulated amyloid deposition affects NSCs behavior and leads to neuronal loss by reducing born and maturation of neurons in SGZ which are vital for learning and memory formation [36]. Although the exact underlying signaling pathway of changed neurogenesis is unclear, several studies have shown that Wnt/β catenin seems to be an important regulator of the SGZ neurogenesis which is downregulated in AD and contributed to cutting down on neurogenesis [20, 37–41]. Moreover, the astrocyte-derived Wnt/β catenin pathway contributes to adult neuroblast proliferation and directs the fate of NSCs into the neural lineage [40, 42]. Intrinsic and extrinsic molecules can modulate the survival of newborn cells to integrate functionally into the circuits of the brain during adult neurogenesis even in a diseased brain [43].

Neurotrophic factor includes various families of proteins that contribute to several cellular processes. Transduction of these factors into the NSCs niche can activate the regulatory mechanism of neurogenesis, synaptic plasticity, and learning and memory improvement [43]. Neurotrophic factors can be transported into the CNS via various pathways including viruses, biomaterials, and ultimately cell-mediated delivery [44]. Plenty of studies have demonstrated that different stem cells mainly MSCs can be employed as neurotrophic factor releasing machines owing to innate NTFs production and secretion abilities [45–49]. Since the neurotrophic factors delivery can facilitate the bioactivity of neurons even in pathogenic conditions, stem cells as a vector for continuous NTFs delivery could be actual therapy for neurodegenerative disorders [50–52]. As previously demonstrated by Jahed et al. NTF-SCs can reduce the AD pathology through down-regulation of hyperphosphorylated Tau protein and up-regulation of synaptophysin in in vitro conditions [26]. Further, the alleviation of neurodegenerative symptoms after transplantation of NTF-SCs has been revealed in other animal models. The reported beneficial effects included successful migration, NTFs secretion, and initiation of the regenerative pathway in the lesion site [27, 29, 53, 54]. The present study focused to investigate the effects of intra-hippocampal transplanted NTFs secreting astrocyte-like cells on the alteration of neurogenesis by modulating the Wnt/β- catenin signaling pathway in an animal model of AD. Our results showed that ADMSCs differentiated and exhibited classic astrocyte phenotype and could express the astrocyte marker GFAP in the cytoplasm. Further, the data of the present study displayed that NTFs secreting astrocyte-like cells increased the production of NGF and BDNF proteins [25, 26] which are the two abundant members of the neurotrophin family in the hippocampus and cortex. They contribute to survival, synaptic plasticity, regenerative processes, and other various cellular functions via activating tyrosine receptors (Trk) [55–58]. Evidence showed that levels of BDNF and NGF reduce during aging and AD-like condition [59], while both NGF and BDNF play a critical role in inducing survival and regeneration of neural cells of aged and AD brains [60]. For induction of the AD model, aggregated Aβ_1-42_ was injected into the lateral ventricle and the data of the passive avoidance test revealed deficiency in learning and memory as have shown in other studies [61–63]. For more assurance about the AD model, data of histological analysis showed that Aβ1-42 injection increased the number of amyloid plaques [64]. Then, NTF-SCs were transplanted into the hippocampus and the special learning and memory, the neurogenesis, and Wnt/β-catenin-related genes were assessed. MWM data revealed that injected amyloid reduced learning and memory. Moreover, the IF results showed a reduction in the number of Nestin and NeuN expressing cells in the AD model. The data of real-time PCR suggested that the amyloid-related neurogenesis reduction was the result of upregulated GSK-3β in concurred with the previous study [42]. The GSK-3β is known as a specific kinase in CNS and is curtail for hippocampal memory formation and axonal elongation moreover, plays role in the dysfunction of CNS. The upregulated GSK-3β adversely affects β catenin via its phosphorylation consequently the un-stabilized β catenin cannot initiate the downstream synaptogenesis or other Wnt/β- catenin-related signaling cascades [65]. The β- catenin contributes in neuroprotective, neuronal surviving, anti-apoptotic and synaptic plasticity pathways and studies revealed that the neurodegeneration prevents the β- catenin activity [66, 67]. Studies also have revealed that the Wnt, the upstream of β-catenin reduced in AD [68] as demonstrated in current research. The results of this research showed that NTF-SCs transplantation could elevate the Wnt/β catenin, PI3K/AKT, and MAPK/ERK and in parallel caused the enhancement of the BrdU/Nestin and BrdU/NeuN expressing cells in an animal model of AD. The enhanced neurogenesis might protect the brain from AD-related neuronal loss. According to our data, the enhanced Wnt/β-catenin and related gene expression following NTF-SCs restrained the Aβ relating to reduced neurogenesis. Indeed, the Aβ link to the cysteine-rich frizzled domain of the Wnt receptor can stop the Wnt/ β- catenin-related signaling cascades which is essential for neurogenesis [41, 69]. Alvarez et al. found that the addition of Wnt into culture medium improved the survival of hippocampal neurons via inhibiting the Aβ neurotoxicity [70]. Oh et al. demonstrated that MSCs transplantation could enhance neurogenesis through Wnt/ β- catenin signaling in AD mouse model [41]. Therefore, regenerating the Wnt/ β- catenin seems to be an important pathway for Aβ-related impaired brain [20]. Regarding the detailed underlying mechanisms, the activation of the Wnt/ β- catenin pathway through Trk may be via LRP6 phosphorylation downstream of the MAPK/ERK pathway [71]. As shown in the present study, MAPK/ERK levels upregulated in parallel to NTF-SCs transplantation. To clear the exact mechanisms between the Wnt/β-catenin and Trk, further studies are required. The grafted Dil-labeled NTF-SCs could express Nestin and NeuN markers after 5 and 35 days following transplantation. Indeed the type 1 of the stem cells in SGZ, the radial glial cells are astrocyte-like stem cells which are potent to self-renew and differentiate as granular and astrocyte cells [72]. These cells can express the three Nestin, GFAP and sex-determining region Y-box 2 (Sox2) markers [73]. Since the NTF-SCs are astrocyte-like GFAP expressing cells might receive the potential of proliferation and maturation as granular cells inside the neurogenic niche of SGZ therefore, could express Nestin as stem cell and NeuN as mature granular markers. These findings suggest that the NTF-SCs could differentiate into neurons inside the hippocampus, and in this declaration, it must be more cautious, and to elucidate and clarify this opinion need more complementary studies. Taken together, our results suggested the modulating effect of NTF-SCs on endogenous adult neurogenesis through Wnt/ β- catenin signaling in Aβ-related neurotoxic environment and would be considered a strategy for future AD treatment.

## Conclusion

As mentioned above, the results of the current study revealed the effects of NTF-SCs on cognition and neurogenesis in Aβ-induced AD mice and the possible signaling pathway, the Wnt/β- catenin. The belief that newly matured neurons integrate into existent circuits of the hippocampus and their regenerative power is pleasant. The NTFs treatment can be valuable for neurogenesis. The NTF-SCs transplantation as a source for more NTFs availability may provide a new promising idea for the treatment of Alzheimer’s disease.

## Data Availability

Data are available from the corresponding author upon reasonable request.

## Abbreviations

DAPI: 4′, 6-Diamidino-2-Phenylindole, Dilactate
AD: Alzheimer's disease
Aβ: Amyloid-beta
bFGF: Basic fibroblast growth factor
BSA: Bovine serum albumin
BDNF: Brain-derived neurotrophic factor
DG: Dentate gyrus
EGF: Epidermal growth factor
ERK1/2: Extracellular signal-regulated kinase
FBS: Fetal bovine serum
GFAP: Glial fibrillary acidic protein
MAPK: Mitogen-activated protein kinase
NGF: Neural growth factor
NSCs: Neural stem cells
NFTs: Neurofibrillary tangles
NTF-SCs: Neurotrophic factor-secreting astrocyte-like cells
PBS: Phosphate-buffered saline
PI3K: Phosphatidylinositol 3-kinase
PDGF: Platelet-derived growth factor
AKT: Protein kinase B
SGZ: Subgranular zone
GSK-3β: Synthase kinase-3β
